# Rapid Detection of Fraudulent Rice Using Low-Cost Digital Sensing Devices and Machine Learning

**DOI:** 10.3390/s22228655

**Published:** 2022-11-09

**Authors:** Aimi Aznan, Claudia Gonzalez Viejo, Alexis Pang, Sigfredo Fuentes

**Affiliations:** 1 Digital Agriculture, Food and Wine Group, School of Agriculture and Food, Faculty of Veterinary and Agricultural Sciences, University of Melbourne, Parkville, VIC 3010, Australia; 2 Department of Agrotechnology, Faculty of Mechanical Engineering and Technology, University Malaysia Perlis, Arau 02600, Perlis, Malaysia

**Keywords:** adulteration, food fraud, electronic nose, near-infrared, sensor, artificial intelligence

## Abstract

Rice fraud is one of the common threats to the rice industry. Conventional methods to detect rice adulteration are costly, time-consuming, and tedious. This study proposes the quantitative prediction of rice adulteration levels measured through the packaging using a handheld near-infrared (NIR) spectrometer and electronic nose (e-nose) sensors measuring directly on samples and paired with machine learning (ML) algorithms. For these purposes, the samples were prepared by mixing rice at different ratios from 0% to 100% with a 10% increment based on the rice’s weight, consisting of (i) rice from different origins, (ii) premium with regular rice, (iii) aromatic with non-aromatic, and (iv) organic with non-organic rice. Multivariate data analysis was used to explore the sample distribution and its relationship with the e-nose sensors for parameter engineering before ML modeling. Artificial neural network (ANN) algorithms were used to predict the adulteration levels of the rice samples using the e-nose sensors and NIR absorbances readings as inputs. Results showed that both sensing devices could detect rice adulteration at different mixing ratios with high correlation coefficients through direct (e-nose; R = 0.94–0.98) and non-invasive measurement through the packaging (NIR; R = 0.95–0.98). The proposed method uses low-cost, rapid, and portable sensing devices coupled with ML that have shown to be reliable and accurate to increase the efficiency of rice fraud detection through the rice production chain.

## 1. Introduction

Food fraud is an expanding global issue and has become a threat to the food industry and consumers’ confidence. The term "fraud" refers to the intention to deceive consumers concerning the content and quality of the products, which is usually driven by increased profit by the food supplier [1]. It aims to reduce costs by altering the original food product with cheaper replacements and using the appealing quality traits of the original product to the customer. Food fraud through adulteration could result in serious health issues and negatively impact consumer trust in the food industry and government agencies [2]. In the rice industry, most fraudulent cases that have been reported regard rice adulteration and the incorrect labeling of the types of rice sold. As summarized by Śliwińska-Bartel et al. [3], a common form of rice adulteration was the mixture of premium and low-quality rice, such as premium basmati from India and Pakistan, jasmine rice from Thailand, and Wuchang rice from China with lower-quality rice. Therefore, inspection by food regulators at different stages of the supply chain is important to fight rice fraud to protect the consumers and the rice industry. 

Recently, there has been growing interest in developing efficient methods that can detect rice adulteration, such as using multispectral imaging, Fourier-transform near-infrared spectroscopy (FT-NIR), gas chromatography–mass spectroscopy (GC–MS), and deoxyribonucleic acid (DNA)-based analysis techniques [4,5,6]. These techniques have shown high reliability in assessing rice adulteration. However, the instruments are costly and require well-trained personnel to perform the data acquisition, analysis, and interpretation [3]. Even though the extraction of DNA fingerprinting is well-known as a robust analysis method; however, it also has significant drawbacks, such as being expensive, tedious, time-consuming, and requiring skilled personnel [7,8]. 

Several studies have developed spectroscopy-based techniques to predict rice adulteration. Some methods use electromagnetic radiation, in which the absorption intensity at the specific wavelength provides the spectral fingerprint of the material. Previously, Wongsaipun et al. [9] used near-infrared (NIR) spectroscopy combined with a chemometric technique to quantify rice adulteration levels in Thai jasmine rice. High predictive accuracies were obtained from calibration models, resulting in the determination of coefficient (R^2^) values up to 0.98. Furthermore, Li et al. [10] have developed a method to detect rice adulteration using terahertz spectroscopy paired with pattern recognition algorithms. These results showed that support vector machine (SVM) algorithms were the best for identifying rice adulteration with 97.3% accuracy. Findings from previous studies have shown convincing evidence for applying the spectroscopy technique to predict rice adulteration. However, the studies used high-cost instruments, making adoption by food safety authorities for rice inspection difficult. Besides, the terahertz spectroscopy technique mentioned requires sample preparation, wherein the rice grains are ground and compressed into a circular-shaped tablet before performing the analysis, making this approach destructive and time-consuming. 

An electronic nose (e-nose) is a gas sensor array system that detects and distinguishes various targeted gases. Compared to GC–MS analysis, an e-nose provides rapid detection to obtain results in a few seconds or minutes. Previous studies in rice have shown the reliability of e-nose applications such as analyzing the volatile compounds in rice [11], distinguishing expired and non-expired rice [12], monitoring rancidity and insect infestation in brown rice [13], detecting fungal infection in jasmine brown rice [14], and identifying moldy rice [15]. In rice adulteration, Udomkun et al. [16] assessed the feasibility of a commercial e-nose paired with principal component analysis (PCA) to identify the degree of adulteration in Thai jasmine rice in storage conditions. The PCA results showed a clear cluster of adulterated rice samples at the beginning of the experiment; however, high overlap was observed between the rice mixtures over the storage period. 

Currently, the NIR spectrometer and e-nose are available as portable devices at low/affordable costs. They can provide quick and reliable results once combined with machine learning (ML) models [17,18,19]. The artificial neural network (ANN) is one of the frequently applied ML algorithms used to develop prediction models because of its capability to deal with complex multitarget and non-linear relationships to solve food and agriculture problems [20,21]. The combination of low-cost sensors and ML models could play a vital role in practical applications to tackle food fraud in local and global markets throughout the entire food chain. This study aimed to develop rice fraud detection methods using low-cost sensing devices (i.e., handheld NIR spectrometer and e-nose sensors) coupled with machine learning models. Findings from this study may offer food regulators efficient tools to perform on-site inspection to detect rice adulteration using portable, low-cost, user-friendly, non-destructive, and rapid methods. 

## 2. Materials and Methods

### 2.1. Samples Description

Table 1 shows the type of rice samples used in the study. All of the rice was obtained from local supermarkets in Australia. Six combinations of authentic rice with potential adulterants were used to prepare the samples for the experiment. These included rice mixtures from (i) different origins (basmati from India and Pakistan; sushi rice from Australia and the USA), (ii) premium with regular rice (Khoshihikari and regular sushi rice), (iii) aromatic with non-aromatic rice (basmati and long-grain rice; Jasmine and long-grain rice), and (iv) organic with non-organic rice. The rice samples were prepared by thoroughly mixing the authentic rice with adulterants at different ratios by weight (total = 100 g) from 0% to 100% adulteration at 10% increments; for example, a rice sample with 10% adulteration consisted of 90% authentic rice (90 g) and 10% adulterant (10 g). 

### 2.2. Near-Infrared Measurement

A handheld NIR spectrometer, microPHAZIR R.X. Analyzer (Thermo Fisher Scientific, Waltham, MA, USA), was used to obtain the NIR fingerprints of the rice samples in absorbance mode at room temperature. The spectrometer measures the NIR spectral range between 1596 to 2396 nm at every 7 to 9 nm interval. The prepared rice sample was transferred into the original packaging of the authentic rice, followed by the NIR measurement of the rice samples obtained through the packaging window of the rice packaging. This step is important for assessing the ability of the handheld NIR spectrometer to detect rice adulteration at different levels without damaging the original packaging during the actual routine inspection. The NIR measurement was performed in triplicate at ten random points on the packaging window (*n* = 330), and a white background provided by the manufacturer was used at each scan to avoid background noise. The calibration procedure was performed before the first measurement and when prompted by the instruments after 10 to 15 scans. The NIR absorbances of the packaging window were deducted from the absorbance values obtained from the measurement to remove the components of the packaging window for further analysis.

### 2.3. Electronic Nose Measurement

A portable e-nose consisting of nine gas sensors developed by the Digital Agriculture, Food and Wine of the University of Melbourne (DAFW-UoM) [22] was used to obtain the sensor readings in three replicates at room temperature. The sensors have different sensitivity to several gases, including MQ3 (alcohol), MQ4 (methene; CH_4_), MQ7 (carbon monoxide; CO), MQ8 (hydrogen; H), MQ135 (ammonia/alcohol/benzene), MQ136 (hydrogen sulfide; H_2_S), MQ137 (ammonia; NH_3_), MQ138 (benzene/alcohol/ammonia), and MG811 (carbon dioxide; CO_2_) (Henan Hanwei Electronics Co., Ltd., Henan, China). A 500 mL glass beaker was filled with the rice sample and shaken five times before the measurement to help the rice release the aroma into the headspace. The e-nose measurement was obtained from the top opening of the glass beaker, as the size of the e-nose (diameter = 92 mm) was designed to fit the beaker. The e-nose was exposed to the rice sample for 60 s to acquire the sensor reading in the headspace. Calibration was conducted between the measurements for 20 s, allowing the sensors to reach the baseline reading. Supervised code developed in Matlab 2021a (Mathworks Inc., Natick, MA, USA) was used to extract the e-nose output signals by dividing the stable signals into ten equidistance subdivisions to get ten means of the voltage output per sensor [23]. 

### 2.4. Statistical Analysis and Machine Learning Modelling

**Figure 1:**
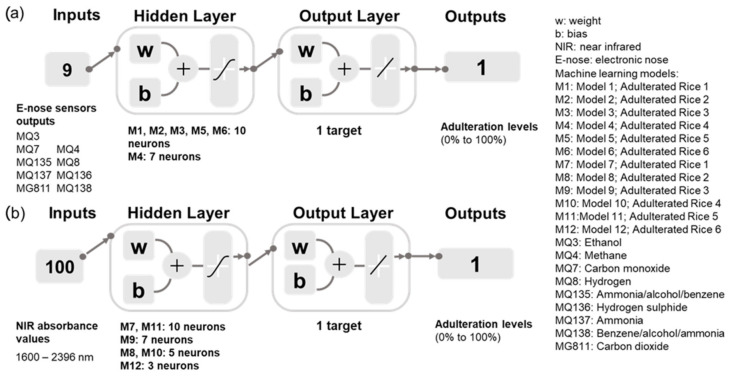
Regression models developed using (**a**) electronic nose sensors data (Model 1–6) and (**b**) NIR absorbance values (Model 7–12) as inputs to predict the rice adulteration levels of six adulterated rice samples. The description of the adulterated rice samples is available in Table 1.

The PCA was used to observe the pattern of the sample distributions on the principal components and their association with the e-nose sensors Matlab 2021a. Six ML models were developed using e-nose outputs (Model 1–6), and another six ML models were constructed using NIR absorbance values (Model 7–12) as inputs to predict the rice adulteration levels based on the regression ANN (Figure 1) using the code developed by the DAFW-UoM group in Matlab 2021a [24]. The models were established by testing 17 algorithms of ANN based on the accuracy, performance from means squared error (MSE), and the lack of signs of the under- or overfitting of the models, followed by a neuron trimming exercise of ten, seven, five, and three neurons [24]. The comparative assessment of these algorithms may obtain the best models to predict rice adulteration levels with the best accuracy and performance. Based on the procedure mentioned above, regression Models 1, 2, 4–6, 10, and 12 were established based on ANN’s Bayesian regularization (BR) algorithm using 70% training and 30% testing data sets. By default, there was no validation data set allocated using the BR algorithm as the regularization step incorporated in the algorithm was used to avoid overfitting [25,26,27]; therefore, the validation process is not necessary to train the model. On the other hand, the Levenberg–Marquardt (LM) algorithm was used to develop Models 3, 7–9, 11, and 12 using 70% training, 15% validation, and 15% testing data division. Figure 1 shows the machine learning models developed in the study to determine rice adulteration levels. Outlier analysis was conducted for all ML models in Matlab 2021a to find the percentage of outliers that may fall within the 95% prediction bounds.

## 3. Results and Discussion

**Figure 2:**
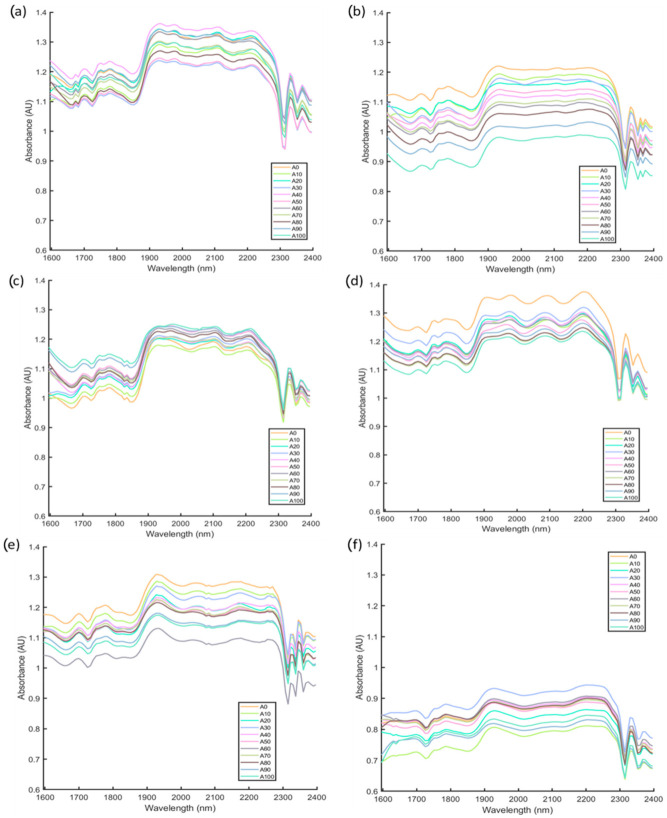
Near-infrared curves of raw absorbance values for adultered rice samples with different proportions of adulterants for (**a**) Adulterated Rice 1, (**b**) Adulterated Rice 2, (**c**) Adulterated Rice 3, (**d**) Adulterated Rice 4, (**e**) Adulterated Rice 5, and (**f**) Adulterated Rice 6. The description of rice mixtures and their abbreviations are shown in Table 1. Abbreviation: 0% adulteration (A0), 10% adulteration (A10), 20% adulteration (A20), 30% adulteration (A30), 40% adulteration (A40), 50% adulteration (A50), 60% adulteration (A60), 70% adulteration (A70), 80% adulteration (A80), 90% adulteration (A90), and 100% adulteration (A100).

Figure 2 shows the raw NIR spectra of the six authentic rice mixed with their possible adulterant at a different ratio by weight. The NIR spectra of the rice showed similar chemical fingerprinting regardless of the proportion of adulteration but with differences in absorbance values. These showed that all rice samples had similar functional groups at different concentrations [28]. The latter could be explained due to the presence of carbohydrates in the form of starch, protein, and lipids as the main components of rice [29]. Strong absorption bands in the NIR region between 1927 nm to 2200 nm were observed in all rice samples, exhibiting a high concentration of carbohydrates (C-H/C=O overtone combination band; 2200–2210 nm), protein (N-H overtone band; 2050–2070 nm), water (O-H; 1940–1950 nm), and lipids (C-H/C=O overtone band; 2140–2150 nm) in rice [30]. Besides, the other overtones observed in this study were the peaks exhibiting bands in the regions 1700 nm (C-H from aliphatic hydrocarbons), 1751 nm (C-H from aromatic hydrocarbons), 1780 nm (O-H from water), 2329 nm (C-H from polysaccharides), and 2367 nm (C-H from aliphatic hydrocarbons) [30]. It can be observed that the absorbance values for Adulterated Rice 6 (Figure 2f) were the lowest compared to other adulterated rice samples. This might be due to the type of rice in Adulterated Rice 6, which was composed of two types of organic and non-organic brown rice that differed from the rest of the rice samples (i.e., white rice) used in this study. 

**Figure 3:**
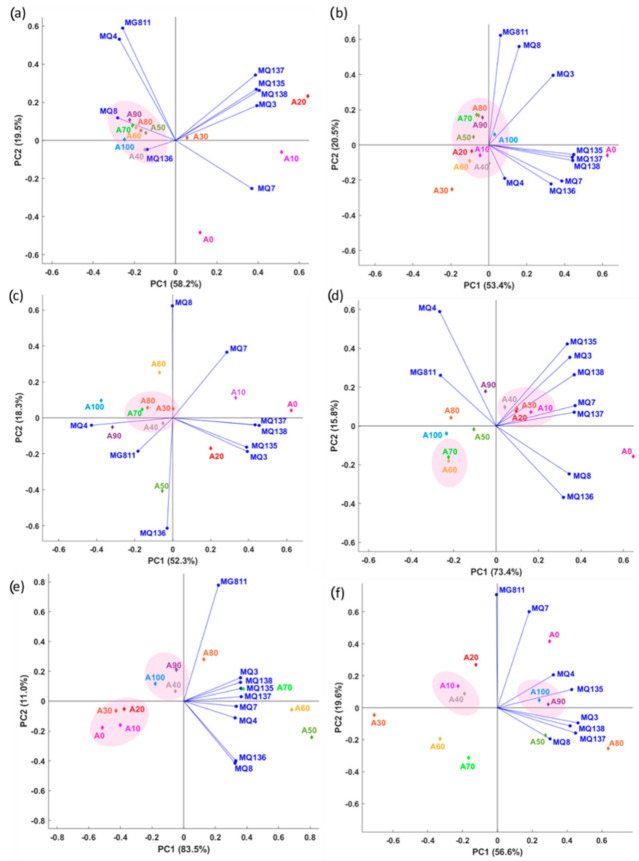
Results from principal components analysis showing a biplot of rice samples with different levels of adulteration and e-nose sensors for (**a**) Adulterated Rice 1, (**b**) Adulterated Rice 2, (**c**) Adulterated Rice 3, (**d**) Adulterated Rice 4, (**e**) Adulterated Rice 5, and (**f**) Adulterated Rice 6. The abbreviations of the rice samples and e-nose sensors are shown in Table 1 and Materials and Methods. PC1: Principle component 1 and PC2: Principal component 2.

Figure 3 shows the first two principal components (PC1 and PC2), in which the total data variability of the rice samples on both PCs is represented by a total of 77.7% (Adulterated Rice 1), 73.9% (Adulterated Rice 2), 70.6% (Adulterated Rice 3), 89.2% (Adulterated Rice 4), 94.5% (Adulterated Rice 5), and 76.2% (Adulterated Rice 6).

For Adulterated Rice 1, PC1 was primarily represented by MQ138 (factor loading: FL = 0.41), MQ3 (FL = 0.39), and MQ135 (FL = 0.39), which explained the separation of A10 and A20 from other samples, while PC2 was presented by MG811 (FL = 0.59) and MQ4 (FL = 0.53). The PC1 of Adulterated Rice 2 was mainly characterized by MQ135 (FL = 0.45), MQ138 (FL = 0.44), and MQ137 (FL = 0.44), while PC2 was characterized by MG811 (FL = 0.62) and MQ8 (FL = 0.56). Adulterated Rice 3 was mainly explained by MQ138 (FL = 0.46) on the positive side and MQ4 (FL = −0.43) on the negative side of PC1. On the other hand, PC2 was mainly explained by MQ8 (FL = 0.62) and MQ136 (FL = −0.61). MQ7 and MQ138 primarily characterized the PC1 of Adulterated Rice 4 and MQ137 with similar FL = 0.37 on the positive side and MG811 (FL = −0.26) on the negative side of PC1. Meanwhile, the PC2 of Adulterated Rice 4 was mainly explained by MQ4 (FL = 0.59) and MQ6 (FL = −0.37). The PC1 of Adulterated Rice 5 was solely characterized on the positive side, mainly by MQ135, MQ137, MQ138, and MQ3 with FL = 0.36, while PC2 was mainly characterized by MG811 (FL = 0.78) on the positive side and MQ8 (FL = −0.42) and MQ136 (FL = −0.40) on the opposite side. Adulterated Rice 6 was solely explained on the positive side of PC1 by MQ3 (FL = 0.46) and MQ137 (FL = 0.45), whereas PC2 was explained by MG811 (FL = 0.71) and MQ7 (FL = 0.60) on the positive side and MQ8 (FL = −0.19) on the opposite side. 

The MQ135, MQ137, and MQ138 sensors were among the highest FL in PC1 of all the PCAs. These sensors are primarily sensitive to ammonia, alcohol, and benzene. On the other hand, MG811 was the sensor sensitive to carbon dioxide and had the highest FL in PC2. These results reflect those of a prior study by Aznan et al. [11], who also found significant positive correlations (*p* < 0.05) between MQ137 and volatile compounds found in raw rice, such as the valeric anhydride (r = 0.53), nonanal (r = 0.49), and octanoic acid (r = 0.54). Octanoic acid is a short-chain fatty acid found in raw rice that was developed through the oxidation of linoleic acid over the storage period [31], while nonanal is one of the major VOCs that contribute to the rice aroma associated with aldehydic, fatty, waxy, citrus, and floral aromas [32]. 

In general, it can be observed that there was no trend of clear separation between most of the adulterated rice samples obtained from the PCA. Minor overlap was observed between A70 and A80 in Adulterated Rice 2 and Adulterated Rice 4 between A20 and A30 and A60 and A70. Besides, groups of rice samples were observed among the rice with a similar percentage of adulteration levels, as illustrated in the circle shown in Figure 3. These results are likely due to the close association in their characteristics among the rice samples with a similar proportion of adulterants. Power et al. [33] and Chen et al. [34] also reported a similar observation in which overlapping samples were observed in PCA plots to indicate a poor discrimination pattern between the samples used in their study. This technique is commonly used among researchers as an unsupervised exploratory data analysis and data dimensionality reduction method [15,35,36,37]. 

The ANN regression models were developed using the e-nose readings (Model 1 to Model 6) obtained from the adulterated rice samples as inputs to predict the rice adulteration levels. The summary of statistical results for the models is shown in Table 2, presenting high accuracies denoted by the correlation coefficient (R) values being close to 1. The first two models were developed to predict the rice adulteration levels of basmati (Model 1; overall R = 0.97) and sushi rice (Model 2; overall R = 0.94) that were adulterated by rice from different origins. Two types of aromatic rice, basmati and Jasmine were mixed with regular long-grain rice as adulterants to obtain input data for the development of Models 3 and 4, respectively. Based on the R-values, both models showed a high predictive ability to obtain adulteration levels in adulterated aromatic rice samples, shown by overall R-values close to 1 (Model 3; R = 0.95, Model 4; R = 0.97). Model 5 was developed to predict premium and regular sushi rice adulteration levels. The model showed high accuracy with an overall R-value of 0.98. Besides, Model 6 was developed to predict adulteration levels in organic rice that had been mixed with non-organic rice. The model also showed high accuracy (R = 0.94). In Table 2, it can be observed that all of the ANN models confirmed no signs of overfitting since the MSE values at the training stage were lower compared to the validation and/or testing stages. Furthermore, comparable MSE values were obtained between the training and testing stages for Models 1–6 and between the validation and testing for Model 3, showing no overfitting signs.

**Figure 4:**
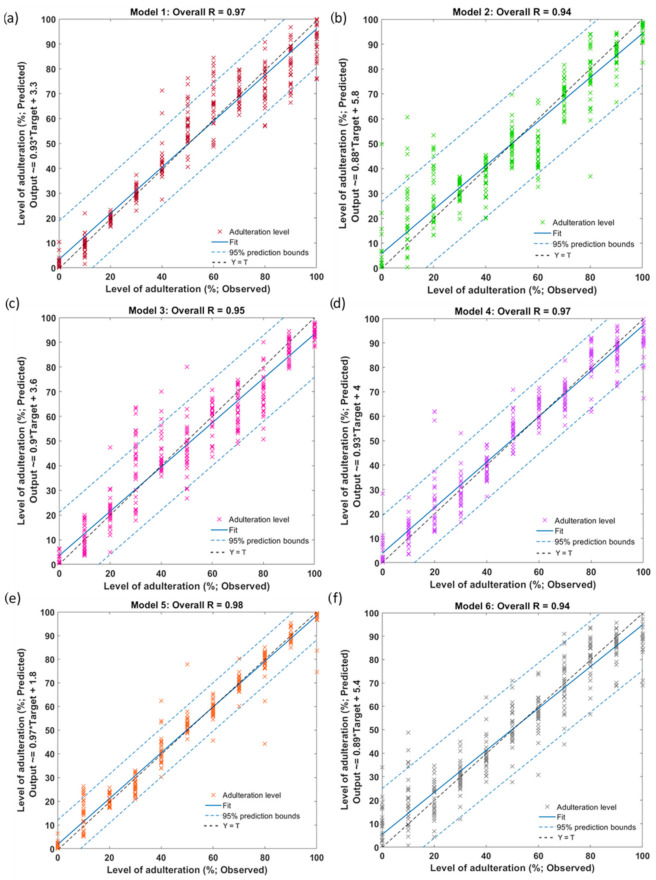
The overall correlation of the regression ANN models developed to predict rice adulteration levels using e-nose sensors for (**a**) Model 1, (**b**) Model 2, (**c**) Model 3, (**d**) Model 4, (**e**) Model 5, and (**f**) Model 6. The abbreviations of the rice samples are shown in Table 1.

The overall regression models to predict the adulteration levels of the rice using e-nose sensor reading as inputs are shown in Figure 4, presenting the 95% prediction bounds with an overall R = 0.94–0.98. The outlier analysis showed that, out of 330 observations, 16 outliers (4.8%) were observed for Models 1–6. Based on the 95% prediction bounds, these results follow the 5% observations expected to fall outside intervals. Furthermore, since the R-values between the targets and outputs of all models are close to 1, the models showed a good fit. 

Table 3 shows the statistical results of the ANN models, including their accuracy, represented by the R-values. Models 7 and 8 were developed to predict adulteration levels in adulterated rice samples from different origins, and both models showed high accuracy with R = 0.96 and 0.98, respectively. The study predicted adulteration levels in aromatic rice samples (Model 9, basmati; Model 10, Jasmine) adulterated with non-aromatic rice. High accuracy was obtained for both models, represented by the overall R = 0.97 and 0.95, respectively. Model 11 was developed to predict adulteration levels in premium sushi rice samples adulterated with regular-grade sushi rice and showed high accuracy (overall R = 0.98). Besides, the ANN model (Model 12) to predict adulteration levels of organic rice mixed with non-organic rice developed in this study also showed high accuracy, represented by the overall R = 0.96.

**Figure 5:**
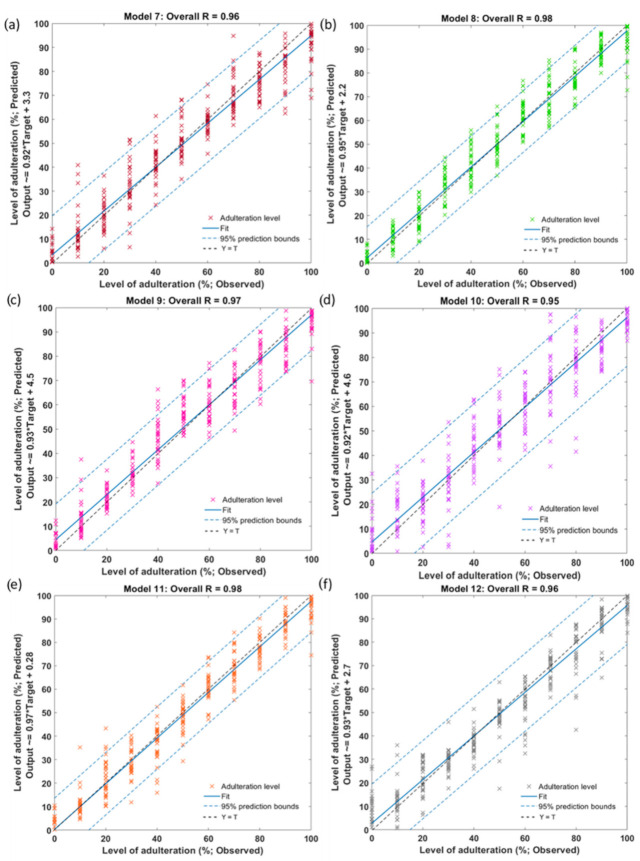
The overall correlation of the artificial neural network regression models developed to predict rice adulteration levels using NIR absorbance values for (**a**) Model 7, (**b**) Model 8, (**c**) Model 9, (**d**) Model 10, (**e**) Model 11, and (**f**) Model 12. The abbreviations of the rice samples are shown in Table 1.

All models showed no overfitting signs described by the lower MSE values obtained at the training stage compared to the validation and/or testing stages. Besides, comparable MSE values were obtained between the validation and testing stages in Models 7, 8, 9, and 11 and the training and testing stages in Models 10 and 12, showing no overfitting signs. Figure 5 shows the overall models, including the 95% prediction bounds for Models 7 to 12 developed using the NIR absorbance values as inputs. Based on the 95% prediction bounds, 4.8% outliers (16 out of 330 observations) were detected in Models 7–12. These results followed the expected 5% observations that may fall outside the prediction interval. Besides, it can be observed that all of the R-values obtained from the models ranging between 0.95–0.98 were close to 1, indicating that the models had a good fit. 

Following the present results, previous studies have shown that NIR spectroscopy and volatile organic compound (VOCs) detection techniques are rapid and non-invasive methods to detect fraud in foods [18,35,38,39] and drinks [33,40]. These include their application developed for fighting rice fraud, noting its popularity for the risk of adulteration along the supply chain [3,9,41]. 

For example, Arslan et al. [42] conducted a recent study to detect rice adulteration in basmati rice using a fabricated colorimetric sensor array system through the discrimination of the authentic and various levels of adulterated rice samples using multivariate statistical analysis. The discrimination results of the k-nearest neighbor (kNN) model developed in the study were highly reliable, showing prediction accuracies of 100% and 95.5% for the calibration and prediction data sets. Despite the high prediction accuracy, obtaining the color-changing profile of the sensor arrays requires the beaker containing the rice sample to be heated in a water bath at 80 °C for 20 min to allow the VOCs to be released in the headspace. Compared to the method proposed in this study, the portable e-nose can obtain sensor responses when exposed to the rice samples as a non-destructive method without requiring any sample preparation, such as heating the samples before the measurement. This highlights the importance of using low-cost sensors, not only in order to decrease the cost but also to reduce the complexity of the detection procedure.

The use of a low-cost and portable NIR spectroscopy technique to discriminate between Thai jasmine rice grown in different regions of Thailand has been recently reported by Srinuttrakul et al. [43]. It is shown that the proposed method is reliable in detecting the geographical origin of jasmine rice samples from two cultivation regions, northern and northeastern, which could be further used as a screening method to detect rice fraud related to geographical origins. However, the developed models using orthogonal projections to latent structures discriminant analysis (OPLS-DA) are limited to qualitatively classifying the two groups of rice from different cultivation regions. 

This study developed a rice fraud detection method using low-cost sensors such as low-cost and portable e-nose sensors and handheld NIR spectrometers. The high accuracies of the ANN regression models obtained in the study suggest that the low-cost e-nose and the handheld NIR spectrometer have the potential to be used as rapid methods to predict various levels of rice adulteration. These include the detection of authentic rice mixed with different proportions of adulterants in the form of similar rice from different origins, aromas, grades, and types of production (e.g., non-organic). The findings have important implications that may help the rice industry to detect rice fraud using a reliable method that is rapid and more economical compared to the conventional approaches (e.g., GCMS and FT-NIR). Moreover, both sensors are portable and do not require sample preparation. Therefore, the tools can be used for on-site applications using the proposed method. Besides, both proposed methods used portable sensing devices that collected the measurements without invasive measurements. Furthermore, the proposed method using the portable NIR spectrometer provides a novelty technique to detect rice adulteration based on non-invasive analysis since the measurement can be obtained from outside the packaging. This may avoid destructive sampling during the inspection. Further research might include more than one type of rice as adulterants since authentic rice in the market might be adulterated with a few types of rice in a single package. 

## 4. Conclusions

This study proposed the rapid detection of rice adulteration levels for various possible combinations of different types of authentic rice and its adulterant using low-cost sensors paired with machine learning models. The study showed that the e-nose sensor and NIR spectrometer are reliable in predicting rice adulteration levels, as shown by the high accuracy of the developed ML models. The findings of this research provide alternative solutions based on low-cost, rapid, and portable identification methods to detect rice fraud due to adulteration of the rice content in the packaging. These results contribute to the rapidly expanding application of digital technologies in the rice industries, which may further benefit consumers to consume high-quality and safe foods. Further work is required to understand better the effectiveness of the e-nose sensor and portable NIR spectrometer performing under different environments to validate its efficiency for application outside the laboratory.

## Figures and Tables

**Table 1:** Details of the adulterated rice samples, including the product category, type of rice, origin, brand name, and abbreviation.

	Product Category	Rice Types	Brand	Abbreviation
Adulterated Rice 1	Basmati	Grown in Pakistan	Riviana	BSR
	Basmati ^1^	Grown in India	Woolworths	BAW
Adulterated Rice 2	Sushi rice	Grown in Australia	SunRice	SRS
	Sushi rice ^1^	Grown in the USA	Pandaroo	PDR
Adulterated Rice 3	Basmati	Aromatic	Riviana	BSR
	Long-grain ^1^	Non-aromatic	Woolworths	LGW
Adulterated Rice 4	Jasmine	Aromatic	Coles	JAS
	Long-grain ^1^	Non-aromatic	Woolworths	LGW
Adulterated Rice 5	Khoshihikari	Premium	Sunrice	KHO
	Sushi rice ^1^	Regular	Sunrice	SRS
Adulterated Rice 6	Medium-grain	Organic	Macro	MOR
	Medium-grain ^1^	Non-organic	Sunrice	MGB

^1^ Rice sample used as an adulterant.

**Table 2:** Statistical results of the artificial neural network regression models developed using electronic nose readings as inputs to predict the rice adulteration levels of the six adulterated rice. Abbreviations: LM: Levenberg–Marquardt; BR: Bayesian regularization; R: correlation coefficient; MSE: mean squared error.

Algorithm	Stages	Samples (n)	Observations (Samples × Target)	R	Slope	Performance (MSE)
Model 1: Adulterated Rice 1						
BR	Training	230	230	0.97	0.95	0.46 × 10^2^
	Testing	100	100	0.95	0.89	1.10 × 10^2^
	Overall	330	330	0.97	0.93	-
Model 2: Adulterated Rice 2						
BR	Training	230	230	0.95	0.89	1.03 × 10^2^
	Testing	100	100	0.90	0.87	1.71 × 10^2^
	Overall	330	330	0.94	0.88	-
Model 3: Adulterated Rice 3						
LM	Training	230	230	0.96	0.90	0.84 × 10^2^
	Validation	50	50	0.95	0.90	1.03 × 10^2^
	Testing	50	50	0.93	0.86	1.09 × 10^2^
	Overall	330	330	0.95	0.90	-
Model 4: Adulterated Rice 4						
BR	Training	230	230	0.97	0.95	0.51 × 10^2^
	Testing	100	100	0.95	0.90	0.96 × 10^2^
	Overall	330	330	0.97	0.93	-
Model 5: Adulterated Rice 5						
BR	Training	230	230	0.99	0.99	0.11 × 10^2^
	Testing	100	100	0.96	0.91	0.69 × 10^2^
	Overall	330	330	0.98	0.97	-
Model 6: Adulterated Rice 6						
BR	Training	230	230	0.96	0.92	0.64 × 10^2^
	Testing	100	100	0.90	0.84	2.10 × 10^2^
	Overall	330	330	0.94	0.89	-

**Table 3:** Statistical results of the artificial neural network regression models developed using the near-infrared absorbance value as inputs to predict rice adulteration levels of the six adulterated rice. Abbreviations: LM: Levenberg–Marquardt; BR: Bayesian regularization R: correlation coefficient; MSE: mean squared error; NIR: near-infrared.

Algorithm	Stages	Samples (n)	Observations (Samples × Targets)	R	Slope	Performance (MSE)
Model 7: Adulterated Rice 1						
LM	Training	230	230	0.98	0.93	0.43 × 10^2^
	Validation	50	50	0.93	0.84	1.47 × 10^2^
	Testing	50	50	0.93	0.91	1.53 × 10^2^
	Overall	330	330	0.96	0.92	-
Model 8: Adulterated Rice 2						
LM	Training	230	230	0.98	0.96	0.44 × 10^2^
	Validation	50	50	0.97	0.94	0.48 × 10^2^
	Testing	50	50	0.97	0.96	0.48 × 10^2^
	Overall	330	330	0.98	0.95	-
Model 9: Adulterated Rice 3						
LM	Training	230	230	0.98	0.95	0.42 × 10^2^
	Validation	50	50	0.95	0.87	0.96 × 10^2^
	Testing	50	50	0.94	0.87	1.07 × 10^2^
	Overall	330	330	0.97	0.93	-
Model 10: Adulterated Rice 4						
BR	Training	230	230	0.97	0.92	0.53 × 10^2^
	Testing	100	100	0.88	0.91	2.36 × 10^2^
	Overall	330	330	0.95	0.92	-
Model 11: Adulterated Rice 5						
LM	Training	230	230	0.99	0.98	0.24 × 10^2^
	Validation	50	50	0.93	0.97	0.90 × 10^2^
	Testing	50	50	0.95	0.97	1.09 × 10^2^
	Overall	330	330	0.98	0.97	-
Model 12: Adulterated Rice 6						
BR	Training	230	230	0.98	0.96	0.28 × 10^2^
	Testing	100	100	0.91	0.87	1.88 × 10^2^
	Overall	330	330	0.96	0.93	-

## Data Availability

Data and intellectual property belong to the University of Melbourne; any sharing needs to be evaluated and approved by the university.
